#  Iranian Version of Manchester Driving Behavior Questionnaire (MDBQ): Psychometric ‎Properties

**Published:** 2016-01

**Authors:** Seyyed Salman Alavi, Mohammadreza Mohammadi, Hamid Soori, Soroush Mohammadi Kalhori, Neda Sepasi, Rasoul Khodakarami, Mojtaba Farshchi, Niloofar Hasibi, Soodabeh Rostami, Hadis Razi, Mohammad Babareisi

**Affiliations:** 1 Psychiatry and Psychology Research Center, Tehran University of Medical Sciences, Tehran, Iran; 2 Safety Promotion and Injury Prevention Research Center, Shahid Beheshti University of Medical Sciences, Tehran, Iran; 3 Young Researchers and Elite Club, Roudehen Branch, Islamic Azad ‎University, Roudehen, Iran, and Psychiatry and Psychology Research Center, Tehran University of Medical ‎Sciences, Tehran, Iran

**Keywords:** Iranian, Manchester Driving Behavior Questionnaire (DBQ), Psychometric Properties, ‎Validity and Reliability

## Abstract

**Objective: **Since the study of driving behavior is of great importance, we conducted this research to ‎investigate the psychometric properties and the factorial structure of the Manchester Driver ‎Behavior Questionnaire (DBQ) in Iranian drivers.‎

**Method:** This cross – sectional research was performed on a sample of 800 drivers (of category D and ‎C) aged 23- 75 who were referred to Imam Sajjad Centre for drug Addiction Diagnosis. ‎Manchester Driver Behavior Questionnaire (DBQ), a demographic questionnaire, were ‎conducted to the sample. To analyze data, we used factor analysis, internal consistency ‎‎(Cronbach's’α), split half, and test-retest using SPSS18 Software.‎

**Results:** As a result of reliability analysis and exploratory factor analysis by principal component and Varimax rotation, we extracted six factors (willful violations, unintentional errors, advertent errors, deliberate mistakes, unintentional violation, and unintentional mistakes, respectively). The factors reliability ranged from 0.65 to 0.75. The test-retest correlations of the DBQ and split- half reliability were 0.56 and 0.77, respectively.

**Conclusion**: The results revealed that the Persian version of the DBQ in category D and C drivers is a ‎valid and reliable tool to assess driving behaviors in Iranian drivers.‎

Driving incidents may cause significant physical damage and high death rate in developing ‎countries (1). Driving accidents, as well as economic problems, can create serious damage to ‎our country (Iran) (2). It has been estimated that every year approximately two million people ‎lose their lives because of road accidents (3), and the number of injured people comes to ‎more than 20 million (approximately 20-50 million) worldwide (4). However, there are some different factors in our country, compared to other countries that increase driving accidents.‎

One of the most important tasks of applied psychologists is to study, comprehend and ‎classify human factors contributing to road accidents (5-6). It should be noted that the term ‎‎“human errors” does not cover all human reasons of driving accidents. In this research, the ‎human factors involved in the occurred accidents were studied, and the results showed that ‎an accurate theoretical framework, for the explanation of why the accidents happen, must ‎discriminate between errors and violations. This view has been exposed by two forms of ‎abnormality, psychological reasons and differential edition methods (7). ‎

Many researchers have proved differentiation between errors and problems in different ‎population (8). Here, error means disability to make sound judgment about the situation and ‎failure to do a series of designed behaviors in order to get good results. (9). Some examples of ‎the behaviors affecting safety of driving include excessive speed or moving without keeping ‎safe distance from other vehicles (8).‎

Based on a reasonable agreement, errors are of two different kinds: The first kind includes ‎errors caused by attention, memory and information processing problems (consisting two ‎major types of slips and lapse); the second kind includes mistakes made by a person selecting ‎inaccurate ways and behaviors to reach his/her aim (without being aware of making mistakes) ‎‎(7). Human reasons of accidents consist of two important types of errors: Unintentional ‎violation including behaviors leading to distraction from rules without any intention such as ‎slow driving on a narrow two-way road; deliberate violation including behaviors done with ‎the purpose of violating the rules and causing damage - considered as destructive behaviors ‎‎(7). However, errors play a specific role as a cognitive dimension and information processing. ‎Also, people with cognitive distortions are more likely to make driving errors (10). Infractions ‎can create special role factors as motivational, social and contextual factors (9). In Iran, some ‎researchers (11-14) have divided human factors of road accident into four groups as the ‎following:‎

1.   Total model of driving including performance problems such as unauthorized speed and ‎inattention to traffic signs, and also inappropriate behaviors such as driving with excess ‎fatigue or carelessness ‎

2.   Perception and sensation errors including low attention, confusion and failure to estimate ‎the distance from other vehicles

3.    Driving under the effect of internal factors, such as consequences of substance abuse, ‎alcohol abuse or a disease ‎

4.    Lack of skills due to low experience and lack of sound judgment

‎Although the role of human factors has been proved to be important in driving accidents (15-‎‎^17^), recognizing human variables (errors or violations), establishing a clear relationship ‎between them and discriminating different types of driving deviations seem difficult (7). ‎

Whereas Iran has the highest rate of driving accidents in the world, in order to assess people ‎mortality rate and economic losses we need tools to identify influential human factors (errors ‎and violations), discriminate them and determine their risk probability. Considering this fact, ‎the current study was conducted to provide a valid and reliable instrument to measure driving ‎behaviors in the study population. Driving behavior questionnaires have been overused ‎around the world, and the questionnaires formal reliability and validity are open to question. ‎Thus, it is important to design and conduct a questionnaire according to the country and the ‎cultural context within which the subjects live (18).‎

## Materials and Method

This research covered a sample of 800 drivers (of category D and C) aged 23- 75 who were referred to Imam Sajjad Centre for drug Addiction Diagnosis. Convenience sampling method was used and it was done at two stages. Questionnaires were distributed to the drivers who had been referred to check their addiction after ensuring that all the drivers regularly used large vehicles. We also checked that all questions were answered. Then, all the drivers were selected to answer all interview items. Inclusion criterion was as follows: Drivers (of category D and C) who were referred to Imam Sajjad Centre; Exclusion criteria were as follows: Female drivers, and illiterate or uneducated drivers who could not understand the questions and refused to complete the questionnaires.

***Manchester Driving Behavior Questionnaire (MDBQ):***

This scale was adjusted and compiled by Rissen et al. in the psychology department of ‎Manchester University (19). It is based on the idea that errors and violations have different ‎psychological reasons and correction methods; hence, they should be discriminated by ‎researchers. Today, MDBQ has been changed into a popular instrument for assessing driving ‎behaviors. This scale has 50 questions with Likert range from 0 to 5. Questions have two ‎different aspects. One aspect is about the kind of behavior, and another relates to amount of ‎risk posed to other drivers. Abnormal behaviors are as follows: Lapse errors, slips, deliberate ‎violation and unintentional violation. These behaviors are classified as follows: 

1.    Behaviors that pose no risk to others, and just give a feeling of comfort (low risk ‎probability)‎

2.    Behaviors that are likely to put others at risk (moderate risk probability)‎

3.    Behaviors that certainly put others at risk (high risk probability)‎

MDBQ has acceptable psychometric properties. Parker and Reason (20) have obtained a ‎correlation coefficient of 0.81 for errors and 0. 75 for violation in reliability research ‎for 80 drivers with a seven-week interval. ‎

For data analysis, we used factor analysis (to analyze construct validity), internal consistency ‎‎(Chronbach’α), split half, and test-retest, respectively. Less than 0.05 were considered to be ‎statistically significant.‎

## Results

***Internal Consistency of the MDBQ:***

First, we calculated the internal consistency for 50 items (Table 1). As the fifth and sixth items had no appropriate intra-class correlation (ICC) with other items, they were omitted from the reliability analysis. Then, we recalculated the reliability of the questionnaire and the results showed a high level of internal consistency for 48 items, suggesting that they were homogenous and none of the 48 items had to be deleted to improve the Cronbach’s α.

***Test –retest and Split-half Reliability***

The test–retest reliability of the questionnaire, administered to 100 drivers with an interval of one month, showed the significance level below 0.001. Consistency of 0.33 between the two administrations was proved by Pearson correlation. Also, the split-half reliability of the questionnaire was ascertained with the correlation coefficient between form I and form II. The correlation was 0.77, and was statistically significant at 0.05 level.

**Table 1:** Reliability Statistics of Manchester Driving Behavior Questionnaire (Cronbach’s Alpha)

	**Mean**	**Std. Deviation**	**Corrected Item-Total Correlation**	**Cronbach's Alpha if Item Deleted**
item.1	16.6512	167.880	0.271	0.864
item.2	15.7294	160.621	0.265	0.867
item.3	16.2648	164.470	0.260	0.865
item.4	15.7250	159.658	0.370	0.863
item.5	16.7713	169.913	0.188	0.865
item.6	16.7120	169.678	0.130	0.866
item.7	16.3343	162.927	0.389	0.862
item.8	16.5398	165.942	0.368	0.863
item.9	16.1129	159.190	0.391	0.862
item.10	16.5181	165.238	0.406	0.862
item.11	16.4834	165.847	0.363	0.863
item.12	16.5557	165.256	0.399	0.862
item.13	16.5123	165.215	0.384	0.862
item.14	15.9421	161.008	0.492	0.860
item.15	16.2938	162.353	0.442	0.861
item.16	16.6151	165.501	0.396	0.862
item.17	16.4370	163.583	0.490	0.861
item.18	16.5369	165.032	0.434	0.862
item.19	16.6093	165.149	0.421	0.862
item.20	16.4906	163.726	0.474	0.861
item.21	16.6237	165.250	0.472	0.862
item.22	16.6483	167.136	0.347	0.863
item.23	16.6165	166.605	0.388	0.863
item.24	16.7554	168.553	0.326	0.864
item.25	16.7207	167.428	0.338	0.863
item.26	16.6512	167.564	0.264	0.864
item.27	16.6729	166.070	0.382	0.863
item.28	16.6802	168.722	0.248	0.864
item.29	16.6194	167.546	0.337	0.863
item.30	16.5499	165.807	0.388	0.862
item.31	16.6802	168.598	0.293	0.864
item.32	16.6744	167.547	0.289	0.864
item.33	15.9899	160.917	0.467	0.860
item.34	16.5470	165.558	0.401	0.862
item.35	16.6006	166.646	0.346	0.863
item.36	16.6946	168.360	0.323	0.864
item.37	16.6628	168.204	0.304	0.864
item.38	16.3140	162.653	0.484	0.860
item.39	15.8828	159.159	0.231	0.872
item.40	16.6368	166.472	0.342	0.863
item.41	16.6208	167.204	0.280	0.864
item.42	16.3097	161.356	0.394	0.862
item.43	16.6643	167.136	0.257	0.864
item.44	16.2214	160.831	0.236	0.869
item.45	15.8828	158.338	0.421	0.861
item.46	16.5123	165.047	0.229	0.866
item.47	16.7077	168.981	0.240	0.864
item.48	16.7959	170.870	0.200	0.865
item.49	16.6454	168.073	0.331	0.864
item.50	16.6483	167.112	0.394	0.863

**Curve 1:**
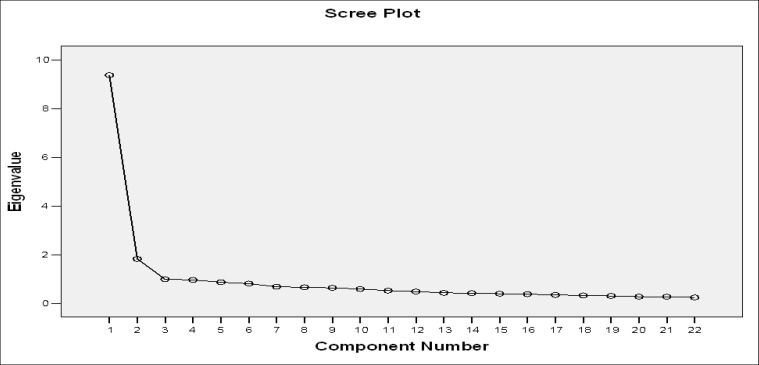
Extracted Factors via Scree Plot

To study the factorial structure of Manchester Driving Behavior Questionnaire, we used exploratory factor analysis and principal properties analysis with varimax rotation. This rotation technique was used based on the assumption that the factors were correlated because the aspects constituting the component (driving behavior) were not independent of each other. For factor analysis, we considered questions with factor loading above 0.4, and Eigen values greater than 1.00 which constitute 52.8% of the total variance.(Curve 1). The Kaiser–Meyer–Olkin (KMO) index was 0.89 for the adequacy of samples (Bartlett’s test of sphericity was significant, df =1128, P<0.0001) and allowed for rejecting the null hypothesis that the variables used in the analysis were not correlated in the studied population. After factor analysis, we obtained the internal consistency for each factor. First factor, “Willful Violations”: This factor covered 8 questions. Questions 16 and 27 had 0.46 and 0.63 minimum and maximum impact factor, ‎respectively. Internal consistency coefficient for this ‎factor was 0.70 (α = 0.70). Second factor, “an ‎Unintentional Error”: This factor included 8 questions; ‎the highest and lowest values of factor loading were ‎‎0.87 and 0.43, respectively. Internal reliability ‎coefficient was 0.72(α = 0.72). ‎

Third factor, “Inadvertent Errors”: This factor covered ‎‎5 items; the maximum and minimum values of impact ‎factor were 0.72 and 0.42, respectively. Reliability ‎coefficient was 0.73 (α = 0.73). Forth factor, deliberate ‎mistakes: This factor consisted of 4 questions; the ‎highest and lowest values of factor loading for these ‎questions were 0.84 and 0.67, respectively. ‎Chronbach’s α of this factor was 0. 65 (α = 0.65). Fifth ‎and sixth factors were “Unintentional Violations” and ‎‎“Unintentional Mistakes”, respectively.‎

Moreover, factor analysis provided support for the ‎construct validity of the questionnaire.‎

## Discussion

Driving behavior is extremely complicated, and none of the existing research methods can ‎cover all the complications. However, Manchester Driving Behavior Questionnaire (MDBQ) ‎is based on a strong theory, and it can differentiate the kinds of drivers’ faults in terms of ‎reasons and risk factors.‎

***Every question of MDBQ has two dimensions:***

The first one determines the nature of the ‎behavior and the other determines the extent of threat posed to other drivers. MDBQ is ‎becoming increasingly a popular and selected instrument to evaluate self-reported driving ‎behaviors. The results of this study revealed that MDBQ questions have acceptable internal ‎consistency and approximately high factor loadings by obvious factor structure. In factor ‎analysis, the six extracted and differentiated factors were: Willful violations (first factor), ‎unintentional errors (second factor), inadvertent errors (third factor), deliberate mistakes ‎‎(forth factor), unintentional violations (fifth factor), and unintentional mistakes (sixth factor). ‎The results of factor structure were consistent with studies carried out by Gras et al. (8), ‎Ozcan et al. (9), Bener (21), Lajunen et al.(22), Berner et al. (23), and Oreizi & Haghayegh ‎‎(24), confirming the reliability and validity of MDBQ. Although in different studies, ‎different factorial structures were obtained, there is consensus on the extracted factors. It ‎seems that these differences are caused by sample size, cultural factors or driver’s category. ‎For example, in the study of Oreizi & Haghayegh, samples had a driving license of category ‎A and in our study samples had a driving license of category D or C. Also, the extracted ‎factors had acceptable internal consistency and significance. The studies of Western, ‎SÂRBESCU, and Haigney & Oreizi were similar (25-26). ‎Therefore, the Persian version of MDBQ administered to drivers of category C and D is ‎based on six main factors. According to these six extracted factors and their inner ‎relationship, we can determine the factorial structure of driving behavior based on scientific ‎literature. ‎

To sum up, the MDBQ has high content and construct validity and can be used to determine ‎driving behaviors of category C and D drivers.‎

Most of the reported correlation coefficients in this study were identical with those obtained ‎from the original questionnaire (24). The other researchers have reported the same correlation ‎coefficients in other countries as well. This finding revealed simplicity and comprehensibility ‎of test phrases in every language such as English, Dutch, Finnish and Persian. Also, the ‎Persian version conformed to the Iranian culture ideally. ‎

## Limitations

Results of this study should be interpreted in the context of its limitations. First, the data in ‎our study were collected from drivers of category D and C; and thus, the results may not be ‎generalized to the drivers of category A or B, motor cycle drivers, or any other groups. ‎Second, we recognized that the data used in this study was cross-sectional, with the level of ‎driving behavior being measured at one point. Third, since there is not the gold standard to ‎measure the cut off, therefore we could not determine the questionnaire cut off and this ‎issue should be examined in the future researches.‎

## Conclusion

The result of this study revealed that MDBQ could evaluate features of driving behaviors of ‎Iranian drivers acceptably. This scale can be used to guide the researches on road accidents. ‎Also, the present study demonstrated that the MDBQ is a self-reported instrument with ‎acceptable psychometric features and content. Providing information about the validity of the ‎DBQ, this study may be highly beneficial to the researchers and road safety practitioners who ‎seek to obtain insight into driving behaviors of a population of interest. 
